# Efficacy and safety of a Chinese herbal formula Maxing Ganshi Decoction in children with community-acquired pneumonia: A randomized, double-blind, placebo-controlled, multicenter trial

**DOI:** 10.3389/fphar.2022.948831

**Published:** 2022-09-02

**Authors:** Yujiao Zheng, Changren Shi, Yaowei Han, Xinmin Li, Lijing Dong, Yan Li, Hui Chen, Yushui Wang, Jinsong Li, Geli Liu, Rong Ma, Fengmei Lian, Xiaolin Tong

**Affiliations:** ^1^ Guang’anmen Hospital, China Academy of Chinese Medical Sciences, Beijing, China; ^2^ College of Traditional Chinese Medicine, Anhui University of Chinese Medicine, Hefei, China; ^3^ The Second Affiliated Hospital and Yuying Children’s Hospital of Wenzhou Medical University, Wenzhou, China; ^4^ First Teaching Hospital of Tianjin University of Traditional Chinese Medicine, Tianjin, China; ^5^ Binhai New Area Hangu Hospital of Traditional Chinese Medicine, Tianjin, China; ^6^ Second Teaching Hospital of Tianjin University of Traditional Chinese Medicine, Tianjin, China; ^7^ Tianjin Hospital of ITCWM Nankai Hospital, Tianjin, China; ^8^ Tianjin Union Medical Center Affiliated to Nankai University, Tianjin, China; ^9^ Tianjin Medical University General Hospital, Tianjin, China

**Keywords:** Chinese medicine, maxing ganshi decoction, community-acquired pneumonia, randomized clinical trial, clinical efficacy

## Abstract

**Background:** As one of the most commonly used Chinese medicine formula in the manage of respiratory diseases, Maxing Ganshi Decoction (MGD) has been demonstrated to improve the clinical symptoms of pneumonia. To evaluate the efficacy and safety of MGD in treating children with community-acquired pneumonia (CAP), we conducted the clinical trial.

**Methods:** A randomized, double-blind, placebo-controlled, multicenter trial was conducted in 3 study sites in Tianjin, China. MDG or placebo were randomly given to patients aged 3–6 years with onset of CAP within 48 h. Changes in disease efficacy during the study period (which was measured as recovery, significant effect, improvement and no effect) was evaluated as the primary outcome. Time from enrollment to fever resolution was assessed as the secondary outcome. The adverse event was analyzed as safety evaluation.

**Results:** A total of 71 patients (36 in MGD and 35 in placebo) were randomized and completed the whole study. The patient demographics and other characteristics at baseline were similar between the 2 groups (*p* > 0.05). After 10 days of intervention, the proportion of recovered and significant effective patients was increased significantly in the MGD group (34.85% [95% CI, 12.44%–57.26%]; *p* < 0.05) compared with the control group. Besides, the symptom score of the MGD group was lowered significantly (*p* < 0.001). The estimated time to fever resolution in the MGD group was also reduced compared with the control group (*p* < 0.05). During the whole study, no side effects were observed in both MGD and control groups.

**Conclusion:** MGD was effective in improving disease efficacy, clinical symptoms and reducing time to fever resolution in patients with childhood CAP, which suggested that MGD may be used as an alternative therapy in the treatment of childhood CAP.

**Clinical Trial Registration:** http://www.chictr.org.cn/showproj.aspx?proj=5612, identifier 13003955.

## Introduction

As a leading cause of mortality and morbidity in children, community-acquired pneumonia (CAP) has brought about serious medical burden both in developing and developed countries, and also become a major public health panic in China (Zar et al., 2017; Zhou et al., 2019). According to the statistics of children under the age of 5 years old, it is estimated that pneumonia resulted in the mortality of approximately 0.921 million children in 2015, and 153.2 per 0.1 million live births die of this disease in China (Liu et al., 2016). Symptoms of childhood CAP include cough or rapid breathing, while chest pain or shortness of breath can also happen. Age, extent of lung involvement, and the organism causing the infection are considered as the infecting factors of symptom severity (Shah et al., 2017). With respect to pathogens, viruses, especially notably respiratory syncytial virus, are the most common cause for CAP children under 5 years old. Among all the pathogenic bacteria induce to childhood CAP, *Streptococcus* pneumonia is the most common bacterial pathogen, other important bacterial causes include Mycoplasma pneumonia and Chlamydophila pneumonia (Leung et al., 2018). For pneumonia caused by bacteria, antibiotics are generally the only choice for the treatment, along with additional supportive and symptomatic treatment. However, the mortality of hospitalized CAP patients is still high despite the application of antibiotic treatment (Restrepo et al., 2008). Besides, with the rising antimicrobial resistance rates and adverse effects of childhood antibiotic use on host microbiome, it is necessary to implement rational antibiotics prescribing as well as seek for other alternative therapies (Tramper-Stranders, 2018).

Traditional Chinese medicine (TCM), which is one of the primary branches of the complementary and alternative medicine, has been implemented in the treatment of respiratory diseases for thousands of years in China and other Asian countries (Xu et al., 2013). As one of the most commonly used TCM formula in the manage of respiratory diseases, Maxing Ganshi Decoction (MGD) has been demonstrated to have obvious efficacy and significantly improve the clinical symptoms of children with pneumonia (Li et al., 2009; Wang et al., 2014; Liu et al., 2019). MGD also named as Maxing Shigan Decoction, which is a classic Chinese herbal formula. The compositons of MGD contain *Ephedra sinica* Stapf, *Prunus armeniaca* L., Gypsum fibrosum, and *Glycyrrhiza uralensis* Fisch. ex DC. A literature study on TCM prevention and treatment of CAP concluded that 16 TCM formulas have been applied in randomized clinical trials (RCT) as well as case series, among which MGD related clinical trials were accounted for most part, indicating that MGD present great potential in the treatment of childhood CAP (Li et al., 2017). However, most of current clinical trials are of low methodological quality, and high-quality RCTs are high required. In order to further evaluate the clinical efficacy and safety of MGD on CAP in children, along with the dose-related response during the whole treatment process, we conducted a randomized, double-blind, placebo-controlled, multicenter trial.

## Materials and methods

### Study design

This study was a prospective, randomized, double-blind, placebo-controlled, multi-center trial between 8 January 2014 to 21 July 2014. It was approved by the Ethics Committee of Guang’anmen Hospital, China Academy of Chinese Medical Sciences (2010EC026-02). Participants were recruited by 3 study sites in Tianjin, China, namely First Teaching Hospital of Tianjin University of TCM, Second Teaching Hospital of Tianjin University of TCM, and Binhai New Area Hangu Hospital of TCM, Tianjin. All legal guardians of every participant signed the written informed consent before enrollment.

### Participant enrollment

Participants enrolled in this study were aged 3–6 years presented with onset of CAP within 48 h and were admitted to hospitals. The study enrollment was based on the following inclusion criteria: 1) They had been diagnosed with childhood CAP [diagnosed clinically and radiologically according to the clinical practice guideline by the Pediatric Infectious Diseases Society and the Infectious Diseases Society of America (Bradley et al., 2011; Ambroggio et al., 2018)] and was in hospital. 2) The body temperature was greater than 37.3°C within 24 h before the first visit. 3) Weight ≥14 kg. 4) Wight blood cells (WBC) ≤ 10 × 10^9^/L and the proportion of neutrophils (NEU%) was less than 70%. 5) C-reactive protein (CRP) was detected as normal (<5 mg/L).

Participants were excluded if they met one or more of the following criteria: 1) They had comorbidities such as heart failure, respiratory failure, toxic encephalopathy, and exudative pleurisy. 2) Definite bacterial infection was detected at the time of enrollment. 3) They had severe primary diseases in heart, liver, kidney and hematopoietic system, or presented with psychiatric illness. 4) They cannot cooperate or were participating in clinical trials of other drugs.

### Drug administration

In this study, the TCM formula MGD was prescribed for the treatment of childhood CAP. MDG originated from a Chinese medical classic named *Treatise on Cold-induced Febride Diseases* in the Han Dynasty (3rd century AD). The composition and dosage of MDG in our study included 4 herbs: *Ephedra sinica* Stapf 6g, *Prunus armeniaca* L. 6g, Gypsum fibrosum 24g, and *Glycyrrhiza uralensis* Fisch. ex DC. 6g, which is listed in Table 1. Herbs used for the decoction and placebo in the controlled group were supplied by Yanjing Herb Pharmaceutical Co., Ltd., (Beijing, China) and were distributed to First Teaching Hospital of Tianjin University of TCM. The herbs were quality controlled in accordance with China Pharmacopoeia (2005 edition) (Commision, 2005). The dosage of herbs was within the safe dosage range specified by the Chinese Pharmacopoeia, and combined with clinical practice experience. Before being applied to the study, all the herbs were examined for heavy metals, microbial contamination, and residual pesticides, and all results have met safety standards in China. Both MGD and placebo were administered in the form of decoction. The preparation of MGD decoction was according to a standardized procedure by a trained technician at First Teaching Hospital of Tianjin University of TCM and distributed to other 2 study sites. The herbs were weighted and soaked into 10 times of the herbs weight of cold water for 1 h, and then boiled the herbs for 50 min, filtrated and concentrated to a final volume of 150 ml decoction. Placebo was prepared according to a standard production process: 5 g cornflour, 16 g brown sugar and an appropriate amount of water were mixed together and boiled for 20 min, filtrated and added 0.005 g sucrose octaacetate to the solution, then concentrated to 1,000 ml and sub-packed in 150 ml for each pack. The appearance and taste sensation of the placebo were similar to the MGD decoction. The MGD and placebo decoction were taken 50 ml orally three times a day after meals.

**TABLE 1:** Composition and dosage of Maxing Ganshi Decoction (MGD).

Name	Chinese name	Part used	Dosage (g)
*Ephedra sinica* Stapf	Mahuang	Dried stems and leaves	6
*Prunus armeniaca* L	Kuxingren	Dried seeds	6
Gypsum fibrosum	Shigao	CaSO_4_ • 2H_2_O	24
*Glycyrrhiza uralensis* Fisch. ex DC.	Gancao	Dried roots and leaves	6

All participants were hospitalized so that they could be quarantined and closely observed and were followed until discharge. Eligible participants were randomized 1:1 by using random-number tables to receive MGD intervention or placebo for 10 consecutive days. The presence and severity of primary symptoms (fever, cough, wheezing, phlegm and pulmonary symptom) and secondary symptoms (thirsty, dry stool, yellow urine, tongue and purse) were recorded daily, which was formulated with reference to TCM Pediatric Disease Syndrome Diagnosis and Curative Effect Standard (State Administration of Traditional Chinese Medicine, 1994). Primary symptoms were scored as 0 (none), 2 (mild), 4 (moderate) and 6 (severe); secondary symptoms were scored as 0 (none), 1 (mild), 2 (moderate) and 3 (severe). The adverse event was also recorded daily. On day 6 and day 10 of the treatment, an all-round efficacy evaluation was performed. Within 10 days, those who had totally relieved from CAP based on efficacy evaluation were out of the group and completed the trial process. However, those who didn’t respond to the treatment (there were no significant changes or exacerbations of symptoms and signs, and the main symptom score decreased by < 33%) can be dropped out of the trial and given another effective treatment.

### Study evaluation and outcomes

The primary outcome was change in disease efficacy during the study period, which was measured as recovery (disappearance of the rales on lung auscultation, complete absorption of inflammation detected by X-ray chest examination, and the reduction rate of symptom score ≥90%), significant effect (disappearance of the rales on lung auscultation, substantial absorption of inflammation detected by X-ray chest examination, and 67% ≤ reduction rate of symptom score <90%), improvement (reduction of the rales on lung auscultation, partial absorption of inflammation detected by X-ray chest examination, and 33% ≤ reduction rate of symptom score <67%) and no effect (no significant changes or aggravation of symptoms and signs, reduction rate of symptom score <33%). The symptom score was measured by the improvement in symptoms including fever, cough, pant, phlegm, lung signs, thirst, dry stool, yellow urine, tough and pulse during the study period. The secondary outcome was time from enrollment to fever resolution (body temperature ≤37.2°C for ≥24 h). The adverse event was analyzed as safety evaluation.

### Statistical analysis

All data were analyzed using the SAS version 8.1 software. Regarding demographic and clinical characteristics at baseline, quantitative variables were reported as mean ± standard deviation (SD), and qualitative variables as frequencies and percentages.

Chi-square test, Fisher exact test or Wilcoxon test was used for assessing quantitative variables, Cochran–Mantel–Haenszel test was used for assessing qualitative variables when considering the influence of multi-center and other factors. For analysis of time from to fever resolution, Kaplan-Meier method was used to estimate the time to resolution when the incidence was 25%, 50% and 75% respectively, and log-rank test was considered to compare the differences between the two groups. A *p* value less than 0.05 was considered statistically significant.

## Results

### Patient characteristics

**FIGURE 1:**
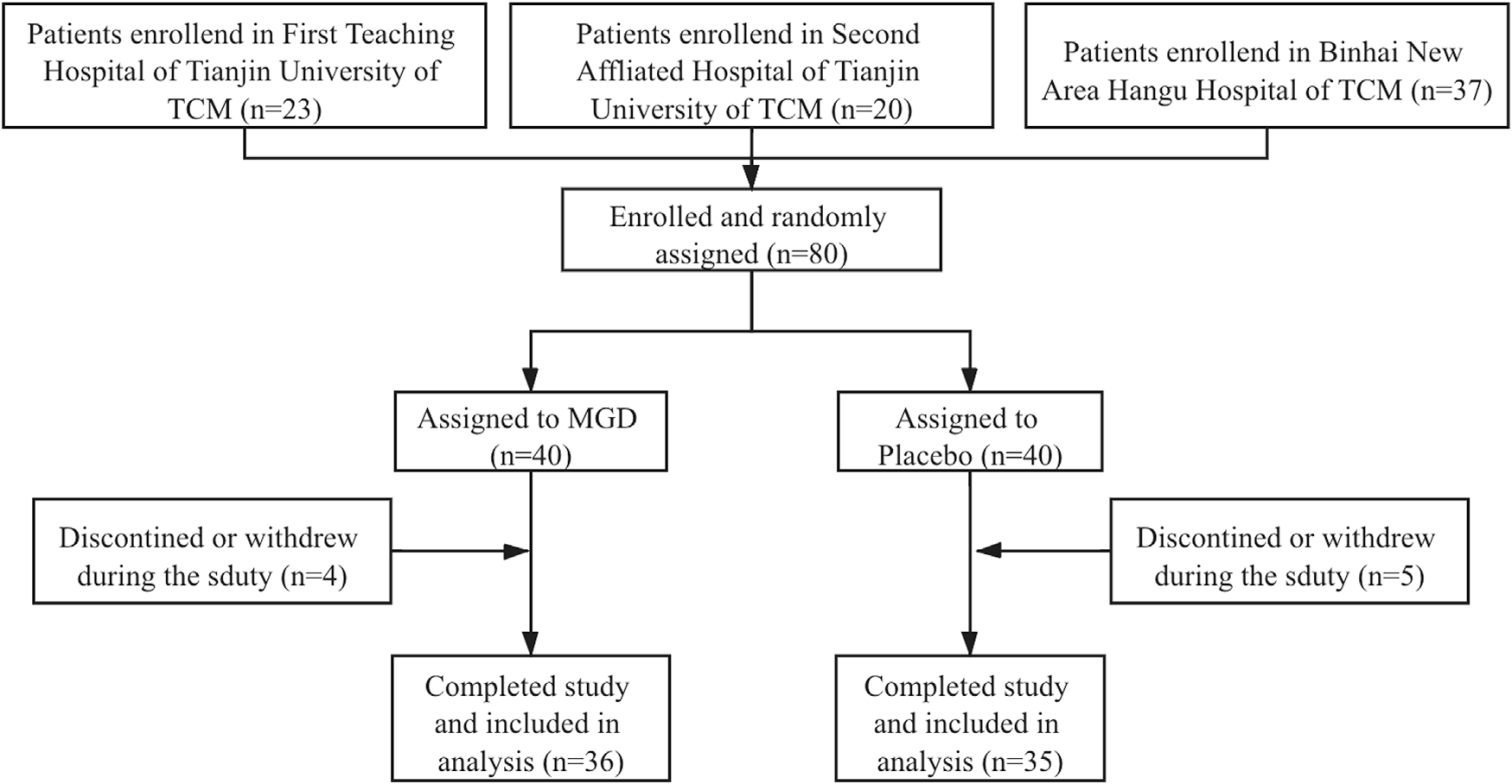
Flowchart of the trial.

A total of 80 patients were enrolled from 3 sites and 71 patients completed the whole study and were randomly assigned to two groups (36 MGD; 35 placebo), 46.48% of the patients were men; the disposition of patients is shown in Figure 1. The patient demographics and other characteristics at baseline were similar between the 2 groups, which is shown in Table 2. No comorbidity or clear bacterial infection were found before enrollment. Mycoplasma infections were identified in both groups (34.29% in the MGD group vs. 34.29% in the control group with clinical significance), and 65.71% of the patients in the MGD group were of no Mycoplasma or clear bacterial infection vs. 62.86% in the control group. During the whole study, the common combined medication included antibiotics, expectorants and antipyretics.

**TABLE 2:** Demographics and baseline characteristics of patients.

Characteristic	MGD (*n* = 36)	Placebo (*n* = 35)	*p* value
Men, n (%)	16 (44.44)	17 (48.57)	0.73
Mean age (SD), y	4.28 (0.96)	4.47 (1.09)	0.45
BMI (SD), kg/m^2^	17.26 (1.99)	18.14 (3.96)	0.59
Body temperature (SD), °C	38.53 (0.81)	38.41 (0.66)	0.71
Course of disease (SD), h	37.00 (14.63)	39.26 (14.16)	0.49
Mean symptom score (SD)	19.67 (3.40)	19.80 (3.19)	0.82
Symptom, n (%)			
Fever	36 (100.00)	35 (100.00)	0.47
Cough	36 (100.00)	35 (100.00)	0.66
Pant	12 (33.33)	13 (37.14)	0.81
Phlegm	36 (100.00)	35 (100.00)	0.38
Lung signs	36 (100.00)	35 (100.00)	0.85
Thirst	34 (94.44)	31 (88.57)	0.25
Dry stool	35 (97.22)	34 (97.14)	0.61
Yellow urine	36 (100.00)	35 (100.00)	1.00
Tough	36 (100.00)	35 (100.00)	1.00
Pulse	36 (100.00)	35 (100.00)	1.00

### Clinical outcomes

Considering changes in disease efficacy, after 6 days of intervention, no patients recovered, 15 patients (41.67%) in the MGD group and 6 patients (17.14%) in the control group showed significant effect. 12 patients (33.33%) in the MGD group showed improvement, while 15 patients (42.86%) in the control group improved; and 9 patients (25.00%) in the MGD group and 14 patients (40.00%) in the control group had no effect. The proportion of recovered and significant effective patients was increased significantly in the MGD group compared with the control group (24.53% [95% CI, 3.30%–45.76%]; *p* < 0.05). After 10 days of intervention, 8 patients (22.22%) in the MGD group and 4 patients (11.43%) in the control group recovered from CAP. 21 patients (58.33%) and 12 patients (34.29%) showed significant effect in the MGD and the control group respectively. 3 patients (8.33%) improved in the MGD group while 14 patients (40.00%) improved in the control group. 4 patients (11.11%) in the MGD group and 5 patients (14.29%) in the control group had no effect. Compared with the control group, the proportion of recovered and significant effective patients was increased significantly in the MGD group (34.85% [95% CI, 12.44%–57.26%]; *p* < 0.05).

The mean baseline symptom score was 19.67 ± 3.40 in the MGD group and 19.80 ± 3.19, which was not statistically significant (*p* > 0.05). After treatment, the symptom score of the MGD group was lowered significantly compared with the symptom score of the control group (*p* < 0.001). For individual symptoms, both two groups were improved significant in fever, cough, pant, phlegm, lung signs, thirst, dry stool, yellow urine, tough and pulse. The comparison between the two groups showed that there was no difference in fever, pant, thirst, dry stool or yellow urine after treatment. Symptoms of cough, phlegm, lung signs, tough and pulse were significantly lowed in the MGD group compared with the control group (Table 3).

**TABLE 3:** Change in symptoms after 6 days and 10 days of intervention.

Symptom, n (%)	MGD (*n* = 36)	Placebo (*n* = 35)	*p* value
Fever (day 6)	0 (0.00)	1 (2.86)	0.56
Fever (day 10)	0 (0.00)	0 (0.00)	0.47
Cough (day 6)	34 (94.44)	34 (97.14)	0.43
Cough (day 10)	14 (38.89)	27 (77.14)	<0.01
Pant (day 6)	1 (2.86)	0 (0.00)	0.63
Pant (day 10)	0 (0.00)	0 (0.00)	0.81
Phlegm (day 6)	33 (91.67)	34 (97.14)	0.80
Phlegm (day 10)	13 (36.11)	29 (82.86)	<0.01
Lung signs (day 6)	35 (97.22)	35 (100.00)	0.17
Lung signs (day 10)	23 (63.89)	30 (85.71)	<0.05
Thirst (day 6)	3 (8.33)	5 (14.29)	0.12
Thirst (day 10)	0 (0.00)	1 (2.86)	0.18
Dry stool (day 6)	19 (52.78)	25 (71.43)	0.10
Dry stool (day 10)	3 (8.33)	14 (40.00)	0.06
Yellow urine (day 6)	15 (41.67)	16 (45.71)	0.72
Yellow urine (day 10)	2 (5.56)	3 (8.57)	0.90
Tough (day 6)	27 (75.00)	35 (100.00)	<0.01
Tough (day 10)	8 (22.22)	2 (62.86)	<0.001
Pulse (day 6)	26 (72.22)	30 (85.71)	0.17
Pulse (day 10)	6 (16.67)	16 (45.71)	<0.01

The effects of the interventions on time from enrollment to fever resolution showed in Table 4. The median time to fever resolution was 0.5 (. to.) in the MGD group and 1.0 (0.5–1.5) in the control group. The 75% incidence of time to fever resolution was 1.0 (0.5–1.5) in the MGD group and 2.0 (1.0–2.5) in the control group. The estimated time to fever resolution in the MGD group was reduced compared with the control group (*p* < 0.05).

**TABLE 4:** Estimates for time to fever resolution.

Kaplan-meier Estimate	MGD (*n* = 36)	Placebo (*n* = 35)	*p* value
25% incidence of time to fever resolution	0.5 (. to.)	0.5 (0.5–1.0)	<0.05
Median time to fever resolution	0.5 (. to.)	1.0 (0.5–1.5)	
75% incidence of time to fever resolution	1.0 (0.5–1.5)	2.0 (1.0–2.5)	

### Safety evaluation

During the whole study, no side effects were observed in both MGD and control groups.

## Discussion

CAP is one of the main health problems in China, and its incidence rate peaks in children younger than 5 years old (Sun et al., 2020b). The results of a cross-sectional study of childhood CAP in China showed that about 99.4% of children received antibiotic treatment, and 23.3% of children received Chinese medicine treatment (Mi et al., 2018). Compared with antibiotic treatment, TCM treatment can get rid of the problem of drug resistance. At present, Chinese medicine is becoming more and more widely used in the clinical application of CAP. On the basis of the effectiveness of TCM treatment, China has established a practical guideline to guide clinical practice. In the guideline, the recommended Chinese medicine prescriptions such as MGD, Yinqiaosan, and Tanreqing injection are all effective at over 65% (Li et al., 2018). In this RCT, we observe the effectiveness of the TCM prescription MGD intervention in children’s CAP, the results found that after MGD intervention, the proportion of recovered and significant effective patients increased significantly compared with the control group. Meanwhile, the symptom score and time to fever resolution were reduced significantly.

Since Chinese medicine is mainly derived from natural plants, animals or minerals, the ingredients are usually complex and diverse, the mechanism of TCM treatments for pneumonia presents a complex feature of multiple targets and multiple pathways. MGD is composed of four Chinese medicines, namely *Ephedra sinica* Stapf, *Prunus armeniaca* L., *Glycyrrhiza uralensis* Fisch. ex DC., and Gypsum fibrosum. A study has found that *Ephedra sinica* Stapf and *Prunus armeniaca* L. are the most important herb pair for treating pneumonia, quercetin, kaempferol, luteolin are the main active ingredients in the herb pair, and the involved treatment mechanisms may include affecting inflammation and immune response, cell apoptosis, hypoxia injury, etc. (Xia et al., 2021). Another study found that the combination of *Ephedra sinica* Stapf and *Glycyrrhiza uralensis* Fisch. ex DC. may exert immune regulation, organ protection and antiviral effects by regulating the PI3K/Akt signaling pathway to treat pneumonia (Li et al., 2021). Scientists have carried out data mining and systematic pharmacology studies on pneumonia, and the results showed that *Ephedra sinica* Stapf, *Prunus armeniaca* L. and *Glycyrrhiza uralensis* Fisch. ex DC. were effective Chinese medicines for the treatment of mycoplasma pneumonia; further analysis of 93 active ingredients in Chinese medicines revealed that TNF, β2AR and PTGS2 played a key role in the anti-pneumonia, and epithelial cell apoptosis (defensive barrier function), GPCR signal transduction (improvement of symptoms) and immune pathways (innate Signal transduction and adaptive Th17 response) were important therapeutic mechanisms (Sun et al., 2020a). One *in vitro* study of MGD on serum of children with Mycoplasma pneumonia also showed that MGD suppressed L-1β, IL-18, TNF-α, down-regulated NLRP3, pro-IL-1β, Caspase-1, pro-Caspase-1, and GSDMD-N in infected cultures and mitigated NLRP3 overexpression-induced pyroptosis (Liu et al., 2021). At present, there are few safety assessments of MGD in the clinical treatment of pediatric pneumonia. In our study, no adverse events have been found at this dose of MGD. However, we need large sample of multicenter clinical trials to systematically evaluate the safety of MGD on vulnerable groups like children, and we also need further research to confirm the safe dose range of MGD for clinical use among children.

Our study has limitations. First of all, if the situations of severe breath shortness, phlegm, high fever and co-bacterial occurred and MGD didn’t work during the study, the use of combined drugs including antibiotics, expectorants and antipyretics were determined by the physician based on the patient’s symptoms, which were anti-pneumonia drugs, thus the efficacy of MGD was difficult to determine and need further vitro and vivo study. Since the use of antipyretic drugs was infrequently (mostly on the first day of treatment), they may have no effect on temperature changes. Secondly, although we only included Mycoplasma-infected CAP at the time of enrollment, a small number of patients were found to have bacterial infections during the intervention process, so antibiotics were used, which may have an impact on our study outcomes.

## Conclusion

In conclusion, our study found that the Chinese medicine MGD can be used as an alternative therapy in the treatment of childhood CAP especially with Mycoplasma infection.

## Data Availability

The original contributions presented in the study are included in the article/Supplementary Material, further inquiries can be directed to the corresponding author.

## Abbreviations

CAP, community-acquired pneumonia; CRP, C-reactive protein; MGD, Maxing Ganshi Decoction; RCT, randomized clinical trials; SD, standard deviation; TCM, traditional Chinese medicine.
